# Can Storage Stability and Simulated Gastrointestinal Behavior Change the Cytotoxic Effects of Concentrated Guava Leaves Extract against Human Lung Cancer Cells?

**DOI:** 10.3390/membranes14050113

**Published:** 2024-05-14

**Authors:** Giordana Demaman Arend, Silvani Verruck, Naira Fernanda Zanchett Schneider, Cláudia Maria Oliveira Simões, Marcus Vinícius Tres, Elane Schwinden Prudêncio, José Carlos Cunha Petrus, Katia Rezzadori

**Affiliations:** 1 Department of Chemical and Food Engineering, Federal University of Santa Catarina, Florianópolis 88040-900, SC, Brazil; giordana.darend@gmail.com (G.D.A.); jpetrus@enq.ufsc.br (J.C.C.P.); 2 Department of Food Science and Technology, Federal University of Santa Catarina, Av. Ademar Gonzaga, 1346, Itacorubi, Florianópolis 88034-000, SC, Brazil; silvani.verruck@ufsc.br (S.V.); elane.prudencio@ufsc.br (E.S.P.); 3 Laboratory of Applied Virology, Department of Pharmaceutical Sciences, Federal University of Santa Catarina, Florianópolis 88035-972, SC, Brazil; nairafzs@gmail.com (N.F.Z.S.); claudia.simoes@ufsc.br (C.M.O.S.); 4 Laboratory of Agroindustrial Processes Engineering—LAPE, Federal University of Santa Maria, Cachoeira do Sul 96503-205, RS, Brazil

**Keywords:** lung carcinoma, cytoprotective effect, *Psidium guajava* L. leaves, phenolic compounds, antioxidant activity

## Abstract

The influence of storage stability and simulated gastrointestinal behavior of different extracts of guava leaves extracts (NC: not concentrated, and C10 and C20: concentrated by nanofiltration) was evaluated based on their total phenolic compound (TPC) contents and antioxidant activity as well as on their cytotoxic effects on A549 and Vero cells. The results showed that C10 and C20 presented high stability for 125 days probably due to their high TPC contents and antioxidant activity. The simulated gastrointestinal behavior modified their TPC contents; however, after all digestion steps, the TPC values were higher than 70%, which means that they were still available to exert their bioactivities. Additionally, the cytotoxic effects of these extracts were evaluated before and after the simulated gastrointestinal behavior or under different storage conditions. C10 presented the best selectivity indices (SI) values (IC50 Vero cells/IC50 A549 cells) at both conditions suggesting that it can be considered a potential extract to be developed as a functional food due to its resistance to the gastrointestinal digestion and storage conditions tested.

## 1. Introduction

Considering the benefits of different extracts of medicinal plants, a proper formulation is essential to ensure their bioaccessibility and resistance to degradation [1]. Membrane processes aiming at the enhancement of bioactive compounds have been widely used due to their intrinsic advantages, such as maintenance of thermolabile compounds, low energy consumption, low processing temperatures, easy operation, and no phase transition [2]. The concentration of plant aqueous extracts using nanofiltration membranes has been widely studied, and the results concluded this is a process with the potential to fractionate and concentrate bioactive compounds [3,4,5,6,7,8].

In many countries, folk medicine has associated the treatment and prevention of several diseases with the consumption of the aqueous extracts of guava (*Psidium guajava* L., Myrtaceae) leaves, which is a tropical America native plant growing in tropical and subtropical areas. In Brazil, guava leaves are widely used for their medicinal properties, and their fruits are consumed in natura or used to produce juice and sweets by the food industry [9]. Phytochemical analyses showed the presence of phenolic compounds as the major bioactive secondary metabolites of this plant, and the reported pharmacological actions include antidiabetic, antihypertensive, anti-inflammatory, anti-allergic, antispasmodic, antimicrobial, antioxidant, antitussive, antigenotoxic, and antiplasmodial activities [9,10,11]. Quercetin, between the phenolic compounds, is identified as the most powerful antioxidant present in guava leaves [12]. 

It is well known that phenolic compounds present poor oral bioaccessibility since only a small portion of these molecules remains available after oral ingestion to be absorbed from the gut into the bloodstream and then be delivered to the appropriate site [13]. In turn, this factor is generally determined by in vitro digestion procedures that evaluate their liberation from the food matrix and conversion during digestion [2]. According to Jara-Palacios et al. [14], it is recommended that the first step to evaluate the possible effects of a bioactive compound is to evaluate its stability during gastrointestinal digestion. Therefore, the knowledge related to the effects of pH changes, digestive enzymes, food matrix, and interaction with other components becomes an important issue to evaluate the bioaccessibility of bioactive compounds [13]. To the best of our knowledge, there are no studies concerning the bioactive phenolic compounds resulting from the gastrointestinal digestion of guava leaves extracts, and this is the first study reporting these effects.

In another context, an important factor that must be emphasized is the exponential growth of cancer over the years worldwide. In 2020, cancer was responsible for almost ten million deaths. Currently, it was estimated that it is responsible for one out of every six deaths in the world. The main risk factors for cancer are tobacco, alcohol consumption, unhealthy diet as well as the lack of physical exercise. The most common types of cancer are breast, colorectal, lung, prostate, skin and stomach [15]. In Brazil, according to the latest update from the National Cancer Institute [16], these rates are in agreement.

Given the importance of these topics, the main goals of this work were using the aqueous guava leaves extract concentrate by nanofiltration and evaluating the cytotoxic effects of the non-concentrated and concentrated extracts, before and after the gastrointestinal behavior simulation. Moreover, the stability of phenolic compounds from guava during room temperature and refrigerated storage was also evaluated to determine the effects of the temperature on the total phenolic compound contents and their cytotoxic effects after such the used storage conditions. 

## 2. Materials and Methods

### 2.1. Aqueous Guava Leaves Extract Production

Guava (*Psidium guajava* L.) leaves were harvested in Biguaçu (latitude, 27°29′39″ S; longitude, 48°39′20″ W; altitude 2 m above sea level), Santa Catarina state, Brazil. The leaves were identified by comparison with the exsiccate of a previously identified species, code FLOR 67263, from the Herbarium FLOR of the Federal University of Santa Catarina, Florianópolis, SC, Brazil. 

Leaves were picked, washed, bleached (90 °C during 3 min followed by an ice bath until total cooling), dried in a forced air oven (FABBE, 171, São Paulo, SP, Brazil) for 3 h at 45 °C, and ground with a knife mill (Marconi, MA-580, Piracicaba, SP, Brazil). The aqueous guava leaves extract was produced according to Arend et al. [8] using the proportion 1:50 (leaves/water, *w*:*v*) at 95 °C, during 10 min, with constant stirring. This extract was filtered using a nylon filter (125 µm pore size), the volume was adjusted with distilled water, and was tagged as the non-concentrated extract (NC).

### 2.2. Nanofiltration Concentration Process

The nanofiltration procedure was performed to concentrate the NC. The process was performed using a tangential system equipped with polyvinylidene fluoride (PVDF) spiral nanofiltration membrane (Osmonics, Minnetonka, MN, USA), with molar mass cut-off with ranges from 150–300 Da, a filtration area of 1.2 m^2^ and feed spacer of 0.7 mm. The membrane has maximum operating pressure of 32.0 Bar, and maximum operating temperature of 50 °C with pH range between 3.0 and 9.0. The concentration was performed in an open system with permeate removal, and the operating parameters were previously determined: temperature of 35 ± 1 °C and pressure of 8.0 Bar, until reaching the volume reduction factors (VRF) of 10 and 20. The samples obtained in this process were named as C10 for the VRF = 10 and C20 for the VRF = 20.

### 2.3. Storage Stability Determination

The stability of the NC was determined at 4 ± 2 °C (refrigeration temperature) and 25 ± 5 °C (room temperature) for 125 days. The samples were stored in individual amber flasks, and every 15 days, a sample was collected and frozen at −16 ± 1 °C until their analysis. The NC was used as a control sample. Samples were evaluated for their content of total phenolic compounds, antioxidant activity, and cytotoxic selective effects on human lung cancer cells (A549 cell line). The samples were named according to their concentration and storage conditions: NCR is the non-concentrated extract under refrigeration, C10R is the concentrated VRF = 10 sample under refrigeration, and C20R is the concentrated VRF = 20 sample under refrigeration. Moreover, NCA represents the non-concentrated extract at room temperature, C10A is the concentrated VRF = 10 sample at room temperature, and C20A is the concentrated VRF = 20 sample at room temperature.

### 2.4. Gastrointestinal Behavior Simulation 

The in vitro simulated gastrointestinal behavior was performed according to Verruck et al. [17]. All enzyme solutions were prepared on the day of analysis and filter-sterilized using a 0.22 µm membrane filter (MF-Millipore, Billerica, MA, USA). For this test, all reagents used were of analytical grade and purchased from Sigma (St. Louis, MI, USA). To simulate the temperature (37 ± 1 °C) and the intensity of the peristaltic movements in each digestive compartment of the human body, a water bath with mechanical agitation (Dist DI950M, Florianópolis, SC, Brazil) was used. The analysis was performed using 1 mL of the samples. For the mouth step, samples were homogenized with 48 µL of an α-amilase solution (100 U·mL^−1^ in 1 mmol·L^−1^ CaCl_2_) for 2 min with constant stirring of 200 rpm. For the stomach stage, the pH values of the samples were reduced to 2.0, and 50 µL of a pepsin solution (25 mg·mL^−1^ in 0.1 mol·L^−1^ HCl) were added equally, and reacted for 90 min with constant stirring of 130 rpm. Then, simulating the duodenum step, the pH values of the samples were raised to 5.0, and incubated with 250 µL of bovine bile salts plus pancreatin solution (2 g·L^−1^ of pancreatin and 12 g·L^−1^ of bovine bile salts in 0.1 mol·L^−1^ NaHCO_3_) for 20 min at 45 rpm. Finally, for the ileum step, the pH values of the samples were raised to 6.5, and incubated for 90 min at 45 rpm. The final volumes were adjusted to 10 mL with distilled water, and the digested samples were stored at −16 ± 1 °C until the analysis. The phenolic recovery percentages were obtained by Equation (1).

% recovery = (GS × 100)/initial(1)

where GS was the phenolic concentration of each sample at the chosen step of the simulated gastrointestinal digestion, and initial was the phenolic concentration at the initial step. 

### 2.5. Total Phenolic Compound Contents Determination

The total phenolic compound (TPC) contents of the samples were determined according to the method of Singleton and Rossi [18], which consisted of the reaction of the extracts with sodium carbonate and Folin–Ciocalteu reagent (Êxodo Cientific, São Paulo, SP, Brazil. The solutions were stored in a dark place for 2 h, and the absorbances were measured at 765 nm on a spectrophotometer (UV-Vis mini-1241, Tokyo, Japan). Distilled water was used as a blank. A calibration curve was prepared with gallic acid (0 to 500 mg·L^−1^) and the results were expressed as gallic acid equivalents (mg GAE·mL^−1^). Water was used as a negative control and Trolox (6-Hydroxy-2,5,7,8-Tetramethylchroman-2-Carboxylic Acid) (Sigma, St. Louis, MI, USA). was used as a positive control.

### 2.6. Antioxidant Activity Determination

The antioxidant activity of the samples was determined by the ABTS (2,2′-azino-bis(3-ethylbenzothiazoline-6-sulphonic acid)) (Sigma, St. Louis, MI, USA) method according to Rufino et al. [19]. The ABTS ethanolic solution and the samples were left in a dark place for 6 min, and the absorbances were measured at 734 nm on a spectrophotometer (UV-Vis mini-1240, Tokyo, Japan) using ethanol as a blank. A calibration curve was prepared with gallic acid (0 to 250 mg·L^−1^) and the results were expressed as gallic acid equivalents (mg GAE·mL^−1^). Water was used as a negative control and Trolox (6-Hydroxy-2,5,7,8-Tetramethylchroman-2-Carboxylic Acid) (Sigma, St. Louis, MI, USA) was used as a positive control.

### 2.7. Cytotoxic Effects Evaluation

Cells: Vero cells (kidney fibroblasts of the African green monkey *Cercopitecus aethiopis*, ATCC: CCL 81) and A549 cells (human non-small cells lung cancer, ATCC: CCL 185) were purchased from ATCC (Manassas, VI, USA) and grown in Eagle’s Minimum Essential Medium (MEM; Cultilab, Campinas, SP, Brazil), supplemented with 10% fetal bovine serum (FBS; Gibco, Carlsbad, CA, USA), and incubated at 37 °C in a humidified 5% CO_2_ atmosphere. Cells were maintained free of antibiotics and were routinely screened for microorganisms including mycoplasma.

Sulforhodamine B assay: to determine the cytotoxic effects of the extracts, the sulforhodamine B (SRB) assay was used [20]. Briefly, A549 and Vero cells were cultured in 96-well plates (1 × 10^4^ cells·well^−1^) for 24 h to obtain confluence. Thereafter, cells were exposed to the extracts for 48 h. After incubation, to fix the cells, 10% trichloroacetic acid (TCA) was added to each well for 60 min. Plates were then washed with water to remove TCA and stained with SRB for 30 min. Afterwards, the plates were washed with 1% acetic acid to remove the unbound SRB, and the protein-bound dye was dissolved in 10 mM Tris-Base [tris(hydroxymethyl) aminomethane] solution. Lastly, the optical densities were read at 510 nm on a microplate spectrophotometer Spectra Max M2 (Molecular Devices, Sunnyvale, CA, USA). IC_50_ values were defined as the concentrations that reduced cell viability by 50% when compared to the untreated controls. The experiment was performed in triplicate. 

### 2.8. Statistical Analyses

All analyses were done in triplicate, and the results were evaluated using the software TIBCO^®^ Statistica™ 13 (Tibco Software Inc., Palo Alto, CA, USA). One-way analysis of variance (ANOVA) or Tukey’s post-test were used to determine significant differences (*p* < 0.05). 

## 3. Results and Discussion

### 3.1. Storage Stability Determination

**Figure 1:**
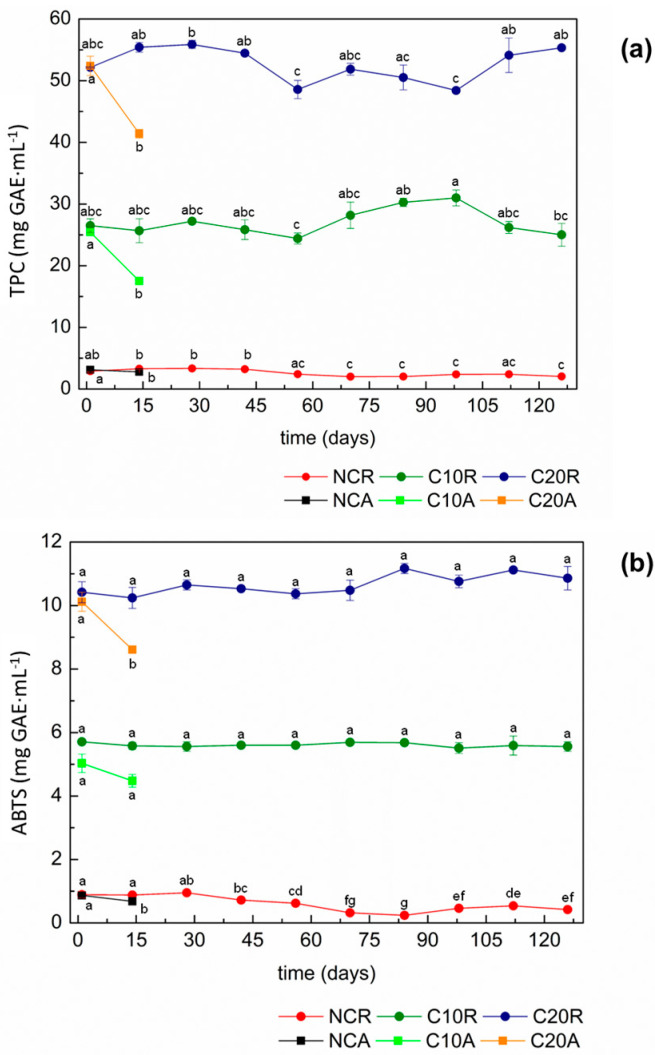
(**a**) Total phenolic compound (TPC) contents and (**b**) antioxidant activity by ABTS method of the non-concentrated (NC) and the concentrated (C10 and C20) guava leaves extracts at refrigerated temperature (R) and room temperature (A) for 125 days. The results are expressed as gallic acid equivalents (mg GAE·mL^−1^). Means followed by the same lower-case letter in the line did not differ statistically at 5% significance.

A long-time stability evaluation of the concentrated guava leaves extracts (C10 and C20) was carried out over a period of approximately four months (125 days) at room temperature (25 ± 5 °C) and refrigeration temperature (4 ± 1 °C) using the NC as a control sample. The results of the determination of TPC contents and the antioxidant activity of these samples are shown in Figure 1.

No statistically significant losses (*p* < 0.05) of TPC were detected under refrigerated storage for the concentrated samples (C10R and C20R). It was expected that all samples had undergone some chemical degradation that would have impacted the TPC contents due to the long storage period. This behavior was detected only for the non-concentrated sample (NCR) and for the FRV 10 sample (C10R), both in cold storage, in which a reduction of approximately 6% and 30% was verified, respectively. 

When stored at room temperature, a maximum reduction of 15% of the TPC contents of the samples has been detected. However, the experiment had to be interrupted after 14 days of storage due to the fungal development on the liquid surface of both non-concentrated (NCA) and concentrated (C10A and C20A) extracts. On the other hand, an increase of approximately 6% of the TPC content of the sample concentrated 20-fold under refrigeration (C20R) was observed. 

Several factors could be related to the different behaviors detected under storage. Zhang et al. [21] have concluded that the TPC contents may be maintained, and in some cases, even enhanced due to the production of new phenolic compounds during the storage time. Tsali and Goula [22] have also reported that during the storage time, the TPC profiles possibly change due to the hydrolysis of conjugated phenolic compounds. Some of these compounds could be degraded, and consequently, new compounds could be formed.

Usually, concentrated extracts present initial high TPC contents and, consequently, a best antioxidant activity. It is well known that the antioxidant compounds in food industry are mainly used to minimize undesirable modifications on the food matrix, which allows the extension of the shelf life of the products [23]. This behavior can be observed when the extract concentration is enhanced and consequently the TPC degradation diminishes, which proves that these compounds act as protective substances. The same trend observed in the present study was observed for other plant extracts. For example, Zhang et al. [21] have detected an increase in TPC content up to 140% for a cranberry juice. Moreno, Cocero, and Rodríguez-Rojo [1] also have detected an increase close to 140% for the grape marc phenolic, with both studies analyzing different storage conditions.

As can be seen in Figure 1b, the antioxidant activity behavior was similar to the TPC contents of the samples tested. The antioxidant activity of the NCR extract was reduced by 52% (*p* > 0.05), but for the C10R extract, this reduction was much smaller (3%), while for the C20R extract, the antioxidant activity increased by approximately 4%. When the samples were exposed to room temperature, the C20A extract reduced the antioxidant activity by 20% at day 15, but as it was described previously, the experiment had to be discontinued due to fungal development.

According to Alzate-Arbeláez et al. [23], the antioxidant activity of TPC present in plant extracts could be unstable in some environmental conditions, such as temperature, light, and presence of oxygen. This activity also depends on their chemical composition since TPC and other secondary metabolites usually bind to double bounds, which can lead to structural changes explaining the different behaviors detected herein. For the refrigerated storage, it was possible to state that the highest concentrations of guava leaves extracts improved the storage stability during the tested period (125 days) due to the maintenance of the TPC contents and the antioxidant activity.

### 3.2. Gastrointestinal Behavior Simulation

**Figure 2:**
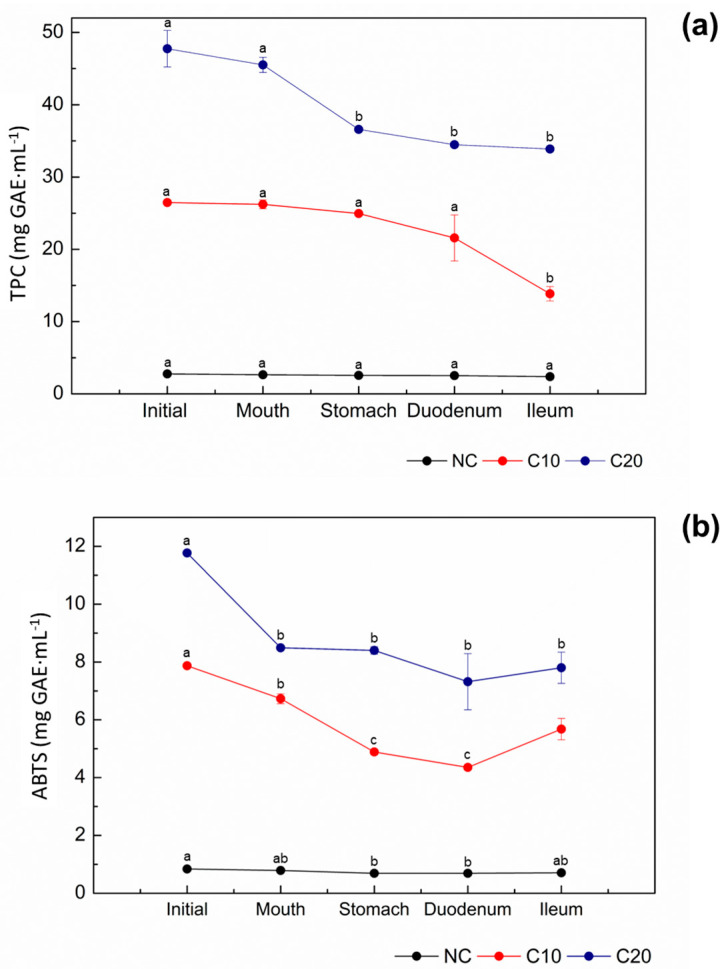
(**a**) Total phenolic compound (TPC) contents and (**b**) antioxidant activity by ABTS method of the non-concentrated (NC) and the concentrated (C10 and C20) guava leaves extracts during the steps of gastrointestinal behavior simulation. The results are expressed as gallic acid equivalents (mg GAE·mL^−1^). Means followed by the same lower-case letter in the line did not differ statistically at 5% significance.

To predict the in vivo behavior of guava leaves extracts, a second experiment was performed, where the samples NC, C10, and C20 stored under refrigeration in a dark place were submitted to a gastrointestinal behavior simulation. The results of the determination of TPC contents and the antioxidant activity of these samples are shown in Figure 2.

For NC, C10, and C20, it was possible to observe a reduction in TPC contents during the gastrointestinal behavior simulation. The TPC contents decreased 7, 6, and 23% for NC, C10, and C20, respectively, after the stomach step (gastric step). For the step that involves duodenum and ileum (intestinal step), the reduction was of approximately 7% for the three samples. Dutra et al. [24] have reported that the TPC contents of the Brazilian fruits (umbu-cajá, siriguela, and mangaba) determined after the stomach and ileum phases were lower than those from the beginning of the gastrointestinal simulation. Similarly, Lucas-González et al. [25] have detected the disappearance of some phenolic compounds at the ileum phase when they studied the flour of persimmon fruits (caqui). All these authors justified this behavior by the fact that they quantify the TPC directly from the matrix, which may have caused changes, such as interactions with other compounds (fibers, proteins, and carbohydrates), chemical reactions (oxidation and polymerization), and structural alterations of the phenolic compounds due to the action of enzymes. Arend et al. [8] evaluated the composition of guava leaves extracts and related that constituents are mainly proteins (0.46 ± 0.05 g⋅100 g^−1^) and carbohydrates (0.50 ± 0.07 g⋅100 g^−1^), corroborating the findings.

The reduction in the TPC contents observed during the stomach, duodenum, and ileum stages, when compared to the initial and mouth steps, can be mainly attributed to the chemical conditions of gastrointestinal digestion, since the structures of these compounds are highly sensitive and can undergo modifications, such as hydrolysis, conversion, and breakdown. So, several factors can be related to the variations detected during the herein simulated gastrointestinal behavior. Thomas-Valdés et al. [26] and Spínola et al. [13] have reported that some factors (oxidation, polymerization, pH variation, interaction with digestive enzymes, pancreatin and bile salts, among others) can be responsible for this behavior. In addition, these authors mentioned that phenolic compounds can also interact with some dietary constituents, such as iron, fibers, pectin, and proteins. Table 1 shows the recovery of TPC (%) from the non-concentrated (NC) and the concentrated (C10 and C20) guava leaves extracts in each step of the gastrointestinal behavior simulation.

The recovery values of TPC showed that the C20 extract was the one which lost most of these compounds (Figure 2a and Table 1). Since this extract presented the highest concentration of TPC in the beginning of the experiment, these compounds are more available in solution and, consequently, were exposed to greater damage caused by enzymes and other compounds.

In relation to the antioxidant activity of NC, C10, and C20 extracts (Figure 2b), it was reduced by 15, 34, and 33% during gastrointestinal digestion, respectively. Several factors can be related to this reduction; however, it is well known that the antioxidant activity correlates directly with the TPC content, which was reduced by 14, 12, and 30%, respectively. This reduction can also be related to the degradation of phenolic compounds that present a relevant antioxidant action. The phenolic compound chemical structures also play an important role in the antioxidant activity and, in this sense, their interactions with other dietary molecules of the food matrix reduce their solubility and, consequently, their antioxidant action [27].

Furthermore, it can be stated that in plant extracts with high contents of TPC, the reduction in these compounds will be also high. For the concentrated extracts C10 and C20, even if they showed a significant reduction in the antioxidant activity, it is possible to point out that 70–80% of their phenolic compounds remained intact after gastrointestinal digestion. So, they were available to be absorbed, distributed, and to exercise their bioactivity. For the concentrated extracts C10 and C20, 71 and 88% of the ingested phenolic compounds remain available to be absorbed and destined for their places of action, respectively. 

Minatel et al. [28] have studied the consumption of phenolic compounds and stipulated that 6.4 to 4.8 mg∙day^−1^ need to be ingested for an effective bioactivity. For the concentrated extract C20, after the gastrointestinal behavior simulation, it was possible to observe a TPC value close to 35 mg∙mL^−1^. Based on this concentration and considering the consumption values recommended by Minatel et al. [28], only 138 mL of the C20 extract would be enough to supply the reported ingestion, demonstrating the efficiency of this extract.

### 3.3. Cytotoxic Effects

Functional foods are frequently related to cancer chemoprevention due to the reduction in the incidence or the proliferation of tumor cells [29]. When samples are screened for their cytotoxic effects on tumor cells, it is also necessary to test these samples concomitantly on non-malignant cells to determine their selectivity indices (SI). In this way, the selectivity index (SI) of a sample is used to determine its selectivity against the tumor cells [30]. The cytotoxic effects of the extracts NC, C10, and C20 before and after gastrointestinal behavior simulation or storage for 125 days on A549 cells (tumoral non-small lung cells) and Vero cells (non-tumoral kidney fibroblasts) are presented in Table 2. 

According to Sul’ain et al. [31] and Braga et al. [29], guava extracts with IC_50_ values below 20–30 µg∙mL^−1^ can be considered to have a significant antiproliferative value. For the tumoral cells A549, the extracts NC, C10, and C20 presented IC_50_ values below 20 µg∙mL^−1^, before and after gastrointestinal simulation or after storage. For the non-tumoral Vero cells, the same extracts presented IC_50_ values ranging from 16.97 to 50 µg∙mL^−1^ before and after gastrointestinal simulation. The selectivity indices (SI) of NC in all conditions, and C10 and C20 extracts after gastrointestinal simulation or after storage are low (from 2.06 to 6.92), which means that they are cytotoxic to tumor cells as well as to non-tumor cells at different levels. Additionally, C10 and C20 with no treatment presented the highest SI values (100.15 and 60.24), respectively. According to Bastos [32], SI values higher than 2 could be considered relevant demonstrating that the sample is twice as active on tumor cells than on non-tumor cells. 

Several reports have shown that the phenolic compounds of guava leaves were able to suppress the proliferation of a wide variety of tumoral cell lines, but the exact mechanism of action has not been elucidated yet [33]. Kawakami et al. [34] have demonstrated that an extract of guava leaves inhibited the proliferation of human colon carcinoma cells, and related that this action occurs by the inhibition of prostaglandin endoperoxide synthase isoforms. Braga et al. [29] have shown the cytotoxic effects of an ethanolic extract of guava leaves on cervical carcinoma (HeLa), colorectal carcinoma (RKO-AS45-1), and lung fibroblasts (Wi-26VA4) cell lines, as well as the reduction of oxidative stress and antioxidant activity. These findings corroborate the results obtained herein, where the lowest IC_50_ values were obtained for the samples with the highest antioxidant activity and the highest TPC content. The last authors explained that these compounds can also inhibit the protein kinases (PK) and the signal transduction of cell proliferation, and consequently the cell cycle is stopped via cyclin-dependent kinases (CDK) by modulating the activity of mitogen-activated PKs (MAPK). Furthermore, the aqueous soluble polyphenolic compounds of guava leaves were related to the induction of apoptotic death of cancer cells by the activation of intrinsic pathways [33].

In addition, during the 125 days storage period, the cytotoxic effects of the NC and the C20 extracts on A549 cells were enhanced, which can be seen through the decrease in their IC_50_ values at approximately 33 and 3 times, respectively, which demonstrated that the storage positively influenced these effects. On the other hand, the IC_50_ value of the C10 extracts was slightly enhanced 1.7 times. After the in vitro gastrointestinal simulation, the cytotoxic effects of NC and C10 and C20 extracts on A549 cells were reduced. This behavior can be verified by the enhancement of their IC_50_ values at 3.5, 15.5, and 3.0 times, respectively, which corroborates the results previously obtained, when the antioxidant activity and the TPC contents were reduced. 

It is important to highlight that even when passing through the gastrointestinal system, when the phenolic compounds reach the ileum, they still present cytotoxic activity. This behavior shows that, even in contact with enzymes and adverse pH conditions, these compounds remain in solution to be subsequently absorbed. In addition, the storage of the samples caused changes in the TPC profiles, improving their cytotoxic activity, and proves that, even if they were stored for a long period of time, these extracts did not lose their effects on A549 cells. The same trend observed herein was reported by Correa et al. [35] in guava leaves extracts on tumoral human cells from the cervix (HeLa), prostate (DU145), mouth (KB), breast (MCF7), colon (HT-29), and leukemia (AML). The authors explained the effects detected by the inhibition of cellular responses as well as the induction of apoptosis. However, no studies were found in the literature that evaluated the cytotoxic effects of guava extracts on A549 cells, demonstrating the importance of the results obtained in this study.

## 4. Conclusions

The results obtained in the present study showed that the concentrated extracts (C10 and C20) presented high stability for 125 days probably due to their high content of phenolic compounds and antioxidant activity. The simulated gastrointestinal behavior modified their TPC contents, but after all digestion steps, the TPC values were higher than 70%, which means that they were still available to exert their bioactivities. 

Additionally, the cytotoxic effects of these extracts were evaluated before and after the simulated gastrointestinal behavior or under the used storage conditions. The C10 extract presented the best SI values in both conditions, suggesting that it can be considered a potential extract to be developed as a functional food due to its resistance to the gastrointestinal digestion and storage conditions tested.

## Figures and Tables

**Table 1:** Recovery percentages of total phenolic compounds (TPC) from the non-concentrated (NC) and the concentrated (C10 and C20) guava leaves extracts in each step of the gastrointestinal behavior simulation.

Step	TPC Recovery (%)		
	NC	C10	C20
Initial	100 ^a^	100 ^a^	100 ^a^
Mouth	96 ^a^ ± 5.0	99 ^a^ ± 1.5	95 ^a^ ± 0.7
Stomach	92 ^a^ ± 5.7	94 ^ab^ ± 0.8	77 ^b^ ± 1.3
Duodenum	92 ^a^ ± 4.6	89 ^b^ ± 1.3	72 ^b^ ± 8.5
Ileum	86 ^a^ ± 4.1	88 ^b^ ± 4.0	71 ^b^ ± 4.9

Means followed by the same lower-case letter in the line did not differ statistically at 5% significance.

**Table 2:** Cytotoxic effects of guava leaves extracts (non-concentrated-NC and concentrated-C10 and C20) on A549 and Vero cells by sulforhodamine B assay, before and after gastrointestinal simulation or storage for 125 days. The results are expressed as IC_50_ (µg∙mL^−1^) and selectivity indices (SI = IC_50_ Vero/IC_50_ A549)).

Extracts	A549 Cells	Vero Cells	SI
	No Treatment		
NC	5.32 ± 1.15	29.46 ± 10.28	5.54
C10	0.27 ± 0.24	27.04 ± 4.64	100.15
C20	0.83 ± 1.01	>50	>60.24
	After 125 days of storage		
NC	0.16 ± 0.23	0.42 ± 0.93	2.62
C10	0.45 ± 0.37	2.10 ± 2.56	4.67
C20	0.26 ± 0.34	0.66 ± 1.39	2.54
	After gastrointestinal simulation		
NC	18.35 ± 30.09	37.80 ± 10.27	2.06
C10	4.17 ± 2.73	28.84 ± 24.83	6.92
C20	2.49 ± 1.22	16.97 ± 10.09	6.81

## Data Availability

The original contributions presented in study are included in article, further inquiries can be directed to the corresponding authors.
